# Patient Satisfaction and Associated Factors among Clients Admitted to Obstetrics and Gynecology Wards of Public Hospitals in Mekelle Town, Ethiopia: An Institution-Based Cross-Sectional Study

**DOI:** 10.1155/2018/2475059

**Published:** 2018-02-01

**Authors:** Taklu Marama, Hinsermu Bayu, Mulualem Merga, Wakgari Binu

**Affiliations:** ^1^Department of Midwifery, College of Health Sciences and Medicine, Wolaita Sodo University, Wolaita Sodo, Ethiopia; ^2^Department of Midwifery, College of Health Sciences and Medicine, Arsi University, Asella, Ethiopia; ^3^Department of Midwifery, College of Health Sciences, Mekelle University, Mek'ele, Ethiopia; ^4^Department of Midwifery, Arba Minch College of Health Sciences, Arba Minch, Ethiopia

## Abstract

**Background:**

To improve the quality of services, satisfying patients is the primary goal of the Ethiopian reform programme.

**Objectives:**

To assess patient satisfaction and associated factors among clients admitted to obstetrics and gynecology wards of public hospitals in Mekelle town. *Method*: Institution-based cross-sectional study design was conducted on 413 participants using systematic sampling methods. Data were collected from March 9 to May 8, 2016, using structured questionnaires. Data were entered and cleaned in Epidata 3.1 and analysed using SPSS V20 with binary logistic regression model. Result. The observed satisfaction rate was 79.7% at 95% CI (75.8%, 83.6%). Clients were dissatisfied towards well-describing side effects of medication, informing what the medication is used for before prescribing and administering, cleanness of toilet and washroom, and access to drinking water, latrine, and hand-washing facility. Respondents live in rural area, stayed < 4 days, admitted for the first time, admitted in Mekelle General Hospital, and who reported their feeling on ways privacy was assured were more likely satisfied than their counterparts.

**Conclusions:**

The observed satisfaction rate is high. So, policymakers, Regional Health Bureau, hospital managers, caregivers, and researchers should plan and work together to keep track of patient satisfaction. Areas patients are dissatisfied should also improve.

## 1. Background

Patient outcomes (70%) and patient satisfaction (30%) measure quality of care. It is important and vital in today's competitive health care environment for patients and the community at large we care about high quality to provide opportunity for improvement such as strategic framing of health plans and searching for ways we can provide them with better service [1–4].

Patient satisfaction survey also plays a vital role in holistic aspects of healing and emotional well-being. First, hospitals that have better engagement with patients may encourage greater observance to clinical standards of care and follow-up; patients who are more satisfied with a service may be more likely to come in for visits and follow the recommendations of the clinicians who they trust. Second, better patient experience scores could indicate that a hospital has stronger teamwork, organizational leadership, and commitment for improvement [5].

Despite a pretty good level of patient satisfaction, a small, but by no means insignificant, proportion of patients expressed dissatisfaction. The fact that patients expressed dissatisfaction with the services indicates that hospital administration needs to do more in the drive towards improving services [6].

Patient satisfaction with hospital care is significantly influenced by patient-provider interactions during the episodes of care, the surrounding physical environment, interpersonal skills in terms of courtesy, respect by health care providers, communication skills, explanation and clear information, and technical skills such as clinical competency and hospital equipment [2, 7].

To improve the quality of services, satisfying patients and clients is the primary goal of the Ethiopian government's reform programme. Community participation is guaranteed that patients' and clients' opinions are heard and their satisfaction with services is optimized through regular surveys on client satisfaction. Hospitals play a crucial role in maternal mortality reduction and should provide quality service to the community and ensure client satisfaction [8, 9].

Hospital is estimated to place at risk an average of $500,000 to $850,000 annually per hospital if patients are dissatisfied. Respondents admitted to obstetrics/gynecology wards were less satisfied than patients admitted to other wards with care services [10–14].

Worldwide, there are varying levels of patient satisfaction. According to a cross-sectional study done on 1847 patients in Pakistan (2015), 90.9% of patients were satisfied with their treatment. Research done in Manipur, India (2007) revealed that 74.1% of patients were satisfied with the overall care received [11, 15].

Patients' dissatisfaction delays institutional skilled birth attendance. According to a mixed study design done in Ethiopia (2013), among 71% of mothers who received antenatal care from a health professional, significant majority (78%) of them were attended by traditional birth attendants at home due to poor quality of care, previous negative experiences, and no attention to privacy and psychosocial support with health facilities. A quantitative objectively observed study (2015) also revealed that there was a high level of disrespect and abuse (78.6%) during childbirth. This will call for 133 million USD/year allowed resource utilization coming through the Millennium Development Goals for health sectors [16–18].

## 2. Methods

The study was conducted in Mekelle town public hospitals, Tigray Region. Mekelle town is located 780 km from Addis Ababa. Mekelle town has 7 subcities (“Kifle Ketema”), 108 “Ketema,” and 60,206 households. There are 4 hospitals, 8 health centers, and 46 urban health extension workers (UHEW) making the health service coverage 56%. Ayder Referral Hospital (ARH) and Mekelle General Hospital (MGH) are the two hospitals selected among four public hospitals purposively.

In this study, an institution-based cross-sectional study design was done. All patients admitted to obstetric and gynecological wards during the study period were included. But, if patients stayed less than 24 hours after admission, if patients were referred to other health institutions with unsuccessful procedures, if patients were seriously ill or unable to communicate or interview, and if patients were treated by sponsors of nongovernment organizations rather than Ethiopian government, they were excluded.

Sample size was calculated using a single population proportion formula considering the assumptions of 50% satisfaction rate [19], 95% level of confidence, *Z*_*α*/2_ = 1.96, and a 5% margin of error (*d* = 0.05):

(1) $$\begin{array}{c} n=\frac{Z_{\alpha /2}^{2}\left(pq\right)}{d^{2}}=\frac{{\left(1.96\right)}^{2}\left(0.5\right)\left(0.5\right)}{0.05^{2}}=384. \end{array}$$

The sample size was 384, and adding for 10% possible nonresponse rate, the total sample size was 423 participants.

A systematic sampling technique was employed to select study participants in the study. The total sample size calculated for the study (423) was distributed to the wards in the town hospitals using proportional allocation based on the estimated number of participants. The first client to be included in the study was selected by lottery method from the first 2 intervals, and then, every 2 participants were included in the study.

Data collection was conducted for two months from March 9, 2016, to May 8, 2016. Six data collectors, with diploma in midwifery, collected data using Tigrinya version translated pretested structured questionnaires. Data were collected as an exit interview after discharge from wards.

Thirty-nine adopted interviewed questionnaires which had four parts were used to collect the data: part I—sociodemographic characteristics of patients (9 items); part II—characteristics of patients (4 items); part III—hospital characteristics; and part IV—satisfaction tools (20 items). The 20 satisfaction tools were categorized into Health Workers' Relationship, Attitude, and Communication (8 items), Health Problem Diagnosis and Management (8 items), and Physical Environment (4 items) aspects. Each tool used to measure the satisfaction rate involved a 5-point Likert scale response (1 = very dissatisfied, 2 = dissatisfied, 3 = neutral, 4 = satisfied, and 5 = very satisfied).

Some words were operationalized as follows:

- Admission mode: the process of patient admission such as either admitted through the emergency department or admitted as scheduled/planned during the liaison.
- Admission ward: the ward where the patient was admitted either as obstetrics ward or gynecology ward regardless of the cause of admission.
- Cause of illness: the reason for the client to visit this hospital categorized as client's perceived to short duration with the disease (acute), perceived to long duration with the disease (chronic), or labour and delivery.
- Dissatisfied: one's below expectation score. Measured as patient/client satisfaction scores below 75% based on satisfaction tool measurements [13].
- Patient satisfaction: experience score of respondents who were admitted to obstetrics and gynecology wards of Mekelle town public hospital and received obstetrics and gynecology-related care during the study period.
- Physical environment: hospital environment including washing facility, toilet, drinking water accessibility, ward accommodation, and cleanness.
- Satisfied: one's expectation score, described as patient/client satisfaction scores above 75% based on satisfaction tool measurements [13].
- Time of hospitalization: it is the time client arrived to the hospital which can be in the morning, evening, or night [20].
- Waiting duration for admission (in days): it is the number of days it takes for the client to be admitted and get a bed [19].

All the collected data were checked for completeness and consistency and then coded and entered into Epidata 3.1.1203.2006 (Epidata Association, Odense, Denmark). Data analysis process was performed using SPSS 20 (IBM Corporation) for windows. Descriptive statistics were described to see the frequency distribution. Frequency distribution tables were used to describe most of the findings. Graph was also plotted for overall satisfaction.

The reliabilities of the variables were checked against the Nunnally's recommended standards (Cronbach's alpha ≥ 0.70). The Cronbach's alpha calculated for all items was 0.89. There was no negative interitem correlation. The 5-point Likert scale satisfaction tool responses were transformed to “satisfied” or “dissatisfied.” Accordingly, the responses of “very satisfied” and “satisfied” were merged as “satisfied,” and the responses of “very dissatisfied,” “dissatisfied,” and “neutral” were transformed into “dissatisfied.” Neutral responses were classified as dissatisfied considering that respondents might represent a fearful way of expressing dissatisfaction. To compute the overall satisfaction rate, mothers who were scored greater or equal to 75% of the 20 tools were considered as “satisfied.”

Bivariate logistic regression model was used to test if there was an association between a dependent variable and each independent variable. Hosmer–Lemeshow model fitness checked was 0.542. Factors statistically significant at *p* value of 0.2 and less at bivariate logistic regression were taken to multivariate logistic regression. Finally, the *p* value of 0.05 and less was considered as statistically significant. The adjusted odds ratio was used to determine the strength of the association between a dependent variable and independent variables.

The questionnaire, information sheet, informed consent, and informed assent were prepared in English and translated into Tigrinya, and Tigrinya was translated back to English to keep its consistency. Data collectors were trained for one day. Close supervision was undertaken during data collection, and every questionnaire was cross checked daily. All the collected data were checked for completeness and consistency.

Ethical approval was obtained from Institutional Review Board (IRB), College of Health Sciences, Mekelle University. Participants  ≥ 18 years old were requested to give their informed verbal consent, and participants < 18 years old were requested to give assent after receiving adequate explanation about title, objective, purpose, procedure, confidentiality, benefit, and risk in participating in this study; the right to withdraw or not to answer questions whenever they felt uncomfortable and who to contact at any time if they have any question were clearly informed to the participants.

Strict confidentiality was assured through anonymous recording and coding of the questionnaires. Participants had given responses in a private room or place in which they felt comfortable after they have finished the discharge process from obstetrics and gynecology wards.

## 3. Results

### 3.1. Sociodemographic Characteristics

Among the 423 sample size, four hundred thirteen mothers (97.6%) admitted to obstetrics and gynecology wards of the public hospital were interviewed. Nearly half (52.6%) of the study population were in the age group of 20–29 years with a median age of 27 years, ranging from 16 years to 60 years. Four-fifths (83.1%) of the participants were married. Majority (79.7%) of the study population was orthodox, and 288 (69.7%) came from urban residential. Regarding occupation, 146 (35.6%) were housewives followed by 131 (31.2%) civil servants. One hundred fifty-three (36.3%) and close to two-fifths (32.2%) of mothers were educated in secondary school, and college and above, respectively. Nearly half (52.8%) of the respondents earned more than 2000 ETB per month. The median household monthly income of the respondents was 2485.00 ETB with minimum 100 ETB and maximum 30,000 ETB (Table 1).

### 3.2. Patient Characteristics

Three hundred ninety-two of the respondents (46.5%) stayed in the wards for less than four days, whereas 124 (30.0%) of them stayed between 4 and 7 days. The rest stayed beyond 7 days. Majority (69.5%) of the respondents were admitted in the hospital for the first time followed by 30.5% repeat. Three-fifths (60.0%) of the participants were admitted for delivery attendance, and 102 (24.7%) and 63 (15.3%) were admitted for acute and chronic case, respectively. Almost half (52.8%) of the respondents were using hospital food.

### 3.3. Hospital Characteristics

Nearly three-fourths (74.8%) of study populations were admitted through emergency. The large majority of the respondents (352, 85.25%) were getting services free of payment. Concerning the time of hospitalization, 196 (47.5%) and 161 (39.0%) of the respondents were admitted in the morning and evening, respectively. Nine-tenths (89.8%) of the respondents reported their feeling on ways privacy was assured (Table 2).

### 3.4. Overall Patient Satisfaction

**Figure 1:**
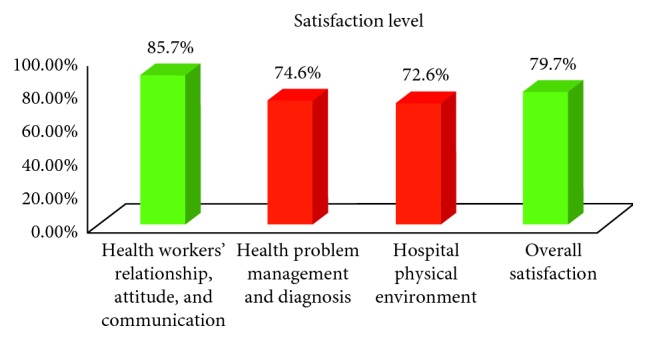
Overall satisfaction rate and aspects of satisfaction tool items of the respondents admitted to obstetrics and gynecology wards of public hospitals in Mekelle town, Tigray Region, 2016.

Overall satisfaction rate was 79.7% at 95% CI (75.8, 83.6). Satisfaction tools have been used in three major aspects. Among seven tools, nine-tenths (85.7%) of the respondents were satisfied with tools used to measure health workers' relationship, attitude, and communication. The rest two aspects are depicted in (Figure 1).

Generally, clients were satisfied with 16 out of 20 tools used to measure patient satisfactions. Clients were satisfied with tools used to measure health care provider's relationship, attitude, and communication which ranges from 86.7% satisfied with health education during discharge on problems to look up to 95.4% satisfied with nurses/midwives being respectful and polite. Mothers were dissatisfied towards well describing side effects of the medication in a way it is understandable; notifying to clients what the medication is used for before prescribing and administering medication; cleanness of toilet and washroom; and access to drinking water, latrine, and hand-washing facility (Table 3).

### 3.5. Factors Associated with Patient Satisfaction among Clients Admitted to Obstetrics and Gynecology Wards of Public Hospitals in Mekelle Town

Among fitted variables included in binary regression model for bivariate analysis, age, residence, duration of stay in the ward, admission status, history of admission, admitted hospital, mothers' privacy, and hospital food were variables taken into consideration for multivariate analysis with *p* value < 0.2. Under multivariate analysis, residence, duration of stay in the ward, history of admission, admitted hospital, and mothers' privacy were found to be statistically significant predictors of patients' satisfaction (Table 4).

Participants who live in rural area were 2.39 times more likely satisfied as compared to participants who live in urban area (AOR = 2.39 at 95% CI = (1.16, 4.92)). Participants who stayed in the ward for less than four days were 56% times more satisfied (AOR = 0.44 at 95% CI = (0.22, 0.88)) as compared to those who stayed for 4–7 days.

Those participants who did not have previous admission were 5.76 times more likely satisfied than who had previously admitted (AOR = 5.76 at 95% CI = (3.17, 10.47)). Mothers admitted in MGH were 2.61 times more likely satisfied than mothers who were admitted to ARH (AOR = 2.61 at 95% CI = (1.23, 5.45)). Participants who report their feeling on ways privacy was assured were 6.32 times more likely satisfied than participants in whom measures were not taken to assure privacy (AOR = 6.32 at 95% CI = (2.78, 14.41)).

## 4. Discussion

In this study, the overall rate of clients' satisfaction was 79.7% at 95% CI (75.8, 83.6). The result is almost similar with studies done in Germany and Asella Hospital, Ethiopia. According to the study done in Germany, 80% of the patients rated the global satisfaction with hospital stay related to all performed services either “excellent” or “good.” In Asella Hospital (Ethiopia), the rate of patient satisfaction was 80.7% [21, 22].

It is low as compared to studies done in Nigeria and India. The satisfaction rate was 96.1%, and 89.1% of the patients were satisfied [6, 23]. In Ethiopia Black Lion Referral Hospital (BLRH) also, the overall patient satisfaction rate was high (90.1%) [14]. This might be related to the characteristics of hospitals studied. The BLRH study specifically focused on nurses, differences in a doctor-patient relationship, and differences in methods. The difference might be due to characteristics of the study population.

However, it is high relative to other studies. According to the Bedford Medical Center, University Hospitals, data, the overall rate of patient satisfaction was 58%. In a Manipur (India) referral institute, the rate of patient satisfaction was 74.1% with hospital care. Besides, in a cross-sectional national exit survey study done in Zambia, the satisfaction rate was 60.9% for non-HIV services and 70.3% for HIV service users [11, 24, 25]. The reason might be due to differences in sociocultural and sample size.

Nationally also, it is lower than the study done in Felege Hiwot Referral Hospital (74.9%) [26], Amhara Region Referral Hospital (61.9%), Eastern Ethiopia Public Hospital (52.7%), and Asella Referral Teaching Hospital (pooled estimate), where the satisfaction rate was 14.7% to 61.5% [27–29]. This might be due to governmental reform implementation on obstetrics and gynecology wards as majority of the studies were of delivery attendant and obstetric emergency.

In this study, participants who live in rural area were 2.39 times more likely satisfied as compared to participants who live in urban area (AOR = 2.39 at 95% CI = (1.16, 4.92)). It is inconsistent with the cross-sectional national exit survey in Zambia in which patients who came from urban area were 53% more likely satisfied as compared to participants who live in rural area [25]. The reason might be due to differences in sociocultural of the study population and sample size in which majority of the Zambia's studies were conducted in health centers.

Conversely, the result is consistent with other studies. In a study done in Uganda, rural delivering mothers were 5.67 times more likely satisfied. In Jimma University Specialized Hospital, respondents coming from rural area were more likely satisfied than those from urban area. In a maternity referral hospital in Ethiopia, mothers who were from the capital city (Addis Ababa) were 2.00 times more likely satisfied with the physical environment [30–32]. This might be due to less expectation from the rural participants because of their previous experience locally where the health facilities might not be of a good standard as of the urban setup or urban residents might have an experience of private clinics that ease for them to compare or access to media might influence their expectation.

According to this study, participants who stayed in the ward for less than four days were 2.27 times more satisfied (AOR = 0.44; 95% CI = (0.22, 0.88)), as compared to those who stayed for 4–7 days. The result is in line with the study done in four Basque hospitals: shorter (<4 days) length of stay showed more satisfaction with visiting and cleanliness [33]. This might be because patients with longer hospital stay were very tired of hospital atmosphere, whereas those stayed for shorter days were quite satisfied with the services provided. Since majority of these cases came through referral, they might be tired of the referral process.

However, it is not consistent with the cross-sectional study done in Iraq in which patients who stayed for longer duration (≥4 days) in the hospital were significantly more satisfied, and a study done in Gandhi Memorial Hospital also displayed that those mothers who stayed above two days in the hospital tended to have higher satisfaction with the health care [32, 34]. This might be due to differences in study population. Participants of the Gandhi Memorial Hospital study were labour and delivery mothers with most of them staying for cesarean section with a fear of other complications as a result of early discharge.

The difference with the study done in Iraq might be that participants were admitted for medical attendance in which majority of them had a history of previous admission that indicates disease condition and experience of hospital admission.

In this study, those participants who did not have previous admission were 5.76 times more likely satisfied than who had previously admitted (AOR = 5.76; 95% CI = (3.17, 10.47)). This study is consistent with the study done in Tuscan Hospitals, Italian Region: those who had no previous admission history were 43% more likely satisfied as compared to patients admitted previously. In Basque Health Care Service on patients admitted to four general hospitals also, those admitted previously showed lower satisfaction with human care, comfort, and cleanliness, and first-time admitted participants were 3.8 times more likely to be satisfied as compared to those who had a history of previous admission according to the eastern Ethiopia public hospitals study [29, 33, 35].

But, incongruent with the study done in Iraq, patients who had a history of previous admission were more satisfied than those who did not have previous admission [34]. This might be due to hospital readmission tiredness/boringness of quality of care during the previous time, or previously admitted mothers might have expected more service than this.

In this study, clients admitted in MGH were 2.61 times more likely satisfied than clients who were admitted in ARH (AOR = 2.61; 95% CI = (1.23, 5.45)). The result is comparable with the study done in Northwest Ethiopia Hospitals, in which patients admitted in referral hospitals were less likely satisfied than patients admitted in general and district hospitals [13]. This could be due to the fact that ARH is a tertiary referral hospital in the region which is admitting by far greater patient flow beyond capacity (8 million people catchment population as compared to 3.5 million people according to Health Sector Development Plan IV), which could hamper the qualities of the health care deliveries in general, and mothers admitted to obstetrics and gynecology wards specifically.

In this study, participants who report their feeling on ways privacy was assured were 6.32 times more likely satisfied than participants in whom measures were not taken to assure privacy (AOR = 6.32; 95% CI = (2.78, 14.41)). This study is reliable with the research done in Amhara Region Public Hospitals, clients who have care providers measure taken to assure privacy during examinations (AOR = 2.1) were significantly associated with maternal satisfaction, and in a cross-sectional study done in Asella Hospital, it is statistically significant that participants who reported privacy respected were 7 times more satisfied than their counterparts [22, 27].

This might be due to mothers who were admitted for reproductive organ-related issues, which is a sensitive organ that a sense of shame is also attached to the process of physical examination, thereby increasing women's discomfort and diminishing their satisfaction levels if privacy is not kept.

## 5. Strengths and Limitations

### 5.1. Methodological Strengths

- Since the interview was made with admitted patients, patients who stay for a long period of time were not missed.
- Participation of patients was also generally good with a 97.6% response rate.

### 5.2. Limitations

- Since patients were interviewed in the hospital setting, they might give responses favoring the care provider resulting in social desirability bias.
- Data collectors were health professionals, which might result in confirmation bias.

## 6. Conclusions

The observed satisfaction rate (79.7%) of clients admitted to obstetrics and gynecology wards of Mekelle town public hospitals is high. Clients are satisfied with the majority of tools used to measure the satisfaction rate. However, concerning hospital staff informing to clients what the medicine was before giving new medicine, describing possible side effects of the medications in ways clients could understand, cleanness of toilet and washroom, and enough drinking water, latrine, and hand-washing facilities are areas in which clients were dissatisfied. Residence, duration of stay in the ward, frequency of admission, admitted hospital, and perceived privacy assured are factors associated with patient satisfaction among clients admitted to obstetrics and gynecology wards of Mekelle town public hospitals. So, policymakers, stakeholders, hospital managers and caregivers, and community leaders should work together on improving the caring environment.

## Figures and Tables

**Table 1:** Sociodemographic characteristics of the respondents admitted to obstetrics and gynecology wards of public hospitals in Mekelle town, Tigray Region, 2016.

Variables	Category	Frequency (*n*)	Percent
Age in years	15–19	26	6.3
	20–24	109	26.4
	25–29	108	26.2
	30–34	91	22.0
	35–39	37	9.0
	>40	42	10.2

Marital status	Married	343	83.1
	Single	48	11.6
	Divorced	12	2.9
	Widowed	10	2.4

Religion	Orthodox	329	79.7
	Muslim	65	15.7
	Protestant	11	2.7
	Other (catholic, Seventh-Day Adventist Church)	8	1.9

Residence	Urban	288	69.7
	Rural	125	30.3

Occupation	Housewife	147	35.6
	Civil servant	129	31.2
	Farmer	64	15.5
	Business man	56	13.6
	Other (daily laborer, student, housemaid, unemployed)	17	4.1

Educational level	No formal education	82	19.9
	1–6 grade	48	11.6
	7–12 grade	150	36.3
	College and university	133	32.2

Household income (per month, in birr)	<1000 birr	83	20.1
	1000–2000 birr	112	27.1
	>2000 birr	218	52.8

**Table 2:** Hospital characteristics of the respondents admitted to obstetrics and gynecology wards of public hospitals in Mekelle town, Tigray Region, 2016.

Variables	Category	Frequency	Percent
Admission mode	Emergency	309	74.8
	Plan	104	25.2

Waiting time for admission (*n* = 105)	<1 day	39	37.1
	1–3 days	34	32.4
	>3 days	32	30.5

Treatment fee	Free	352	85.2
	Paying	61	11.8

Admitted hospital	ARH	279	67.6
	MGH	134	32.4

Admission ward	Obstetrics	258	62.5
	Gynecology	155	37.5

Time of hospitalization	Morning	196	47.5
	Evening	161	39.0
	Night	56	13.6

Respondents' feeling report on ways privacy was assured	Yes	371	89.8
	No	42	10.2

**Table 3:** Satisfaction rate of each tool used to measure the overall satisfaction of clients admitted to obstetrics and gynecology wards of public hospitals in Mekelle town, Tigray Region, 2016.

Components	Satisfied, *n* (%)	Dissatisfied, *n* (%)	Mean ± SD
*Health workers*' *relationship, attitude, and communication*			
Nurses/midwives being respectful and polite	392 (94.9)	21 (5.1)	4.45 ± 0.74
Doctors treat with courtesy and respect	385 (93.2)	28 (6.8)	4.49 ± 0.79
Nurses/midwives explain well and listen carefully to clients	387 (93.7)	26 (6.3)	4.42 ± 0.81
Doctors listen carefully to clients	382 (92.5)	30 (7.3)	4.46 ± 0.87
Doctors explained things in a way the client could understand	377 (91.3)	36 (8.7)	4.52 ± 0.72
Nurse/midwives make adequate visits and responding to patients' calls and requests	374 (90.6)	39 (9.4)	4.46 ± 0.68
Health education during discharge	358 (86.7)	55 (13.3)	4.13 ± 0.10
*Health problem diagnosis and management*			
Perceived competency of doctors	390 (94.4)	23 (5.6)	4.49 ± 0.64
Nurses/midwives' on-time medication administration	379 (91.8)	34 (8.2)	4.43 ± 0.80
Perceived competency of nurses/midwives	371 (89.8)	42 (10.2)	4.50 ± 0.73
Information about procedure and exam	356 (86.2)	57 (13.8)	4.24 ± 1.13
Client's pain well controlled	341 (82.6)	72 (17.4)	3.93 ± 1.00
Access to prescribed medication in the hospital	318 (77.0)	95 (23.0)	4.08 ± 1.04
Before giving new medicine, the hospital staff told to clients what the medicine was	254 (61.5)	159 (38.5)	3.59 ± 1.22
Possible side effects of the medications well described in a way clients could understand	249 (60.3)	164 (39.7)	3.47 ± 1.18
*Physical environment*			
Admission procedure of this hospital	392 (94.9)	21 (5.1)	4.47 ± 0.70
Perceived accommodation of the room	385 (93.2)	28 (6.8)	4.39 ± 0.72
Cleanness of room and bed	369 (89.3)	44 (10.7)	4.30 ± 0.81
Cleanness of toilet and washroom	270 (65.4)	143 (34.6)	3.63 ± 1.21
Access to drinking water, latrine, and hand-washing facility	261 (63.2)	152 (36.8)	3.62 ± 1.15

**Table 4:** Factors associated with patient satisfaction among clients admitted to obstetrics and gynecology wards of public hospitals in Mekelle town, Tigray Region, 2016.

Variable	Overall satisfaction		COR (95% CI)	AOR (95% CI)
	Satisfied	Dissatisfied		
*Age in years*				
15–19	20	6	1.00	—
20–24	94	15	1.90 (0.65, 5.44)	—
25–29	79	29	0.81 (0.30, 2.24)	—
30–34	71	20	1.07 (0.38, 3.01)	—
35–39	27	10	0.81 (0.25, 2.60)	—
>39	38	4	2.85 (0.72, 11.29)	—
*Residence*				
Urban	219	69	1.00	1.00
Rural	110	15	2.31 (1.26, 4.22)^∗^	2.39 (1.16, 4.92)^∗∗^
*Admission mode*				
Emergency	251	58	1.00	
Plan	78	26	0.69 (0.41, 1.18)	
*Duration of stay*				
1–3 days	168	24	1.00	1.00
4–7 days	89	35	0.36 (0.20, 0.65)	0.44 (0.22, 0.88)^∗∗^
>8 days	72	25	0.41 (0.22, 0.77)	0.64 (0.30, 1.34)
*Frequency of admission*				
First time	257	30	6.43 (3.83, 10.78)^∗^	5.76 (3.17, 10.47)^∗∗^
Repeat	72	54	1.00	1.00
*Admitted hospital*				
ARH	206	73	1.00	1.00
MGH	123	11	3.96 (2.02, 7.76)^∗^	2.61 (1.23, 5.45)^∗∗^
*Privacy assured*				
No	16	26	1.00	1.00
Yes	313	58	8.77 (4.43, 17.36)^∗^	6.32 (2.78, 14.41)^∗∗^
*Consuming hospital food*				
Yes	183	35	1.00	1.00
No	146	49	0.57 (0.35, 0.92)^∗^	0.89 (0.46, 1.73)

^∗^ *p* value < 0.05 at bivariate logistic regression; ^∗∗^ *p* value < 0.05 at multivariate logistic regression.
